# Supplementary material for: Impact of Dietary Sodium Butyrate and Salinomycin on Performance and Intestinal Microbiota in a Broiler Gut Leakage Model
Source: Animals (Basel). 2022 Jan 4;12(1):111. doi: 10.3390/ani12010111 (PMC8749775; doi:10.3390/ani12010111)
Supplement: Supplementary file 1 [file animals-12-00111-s001.zip › Supplementary Figure S1_Histogram of read length.pdf]

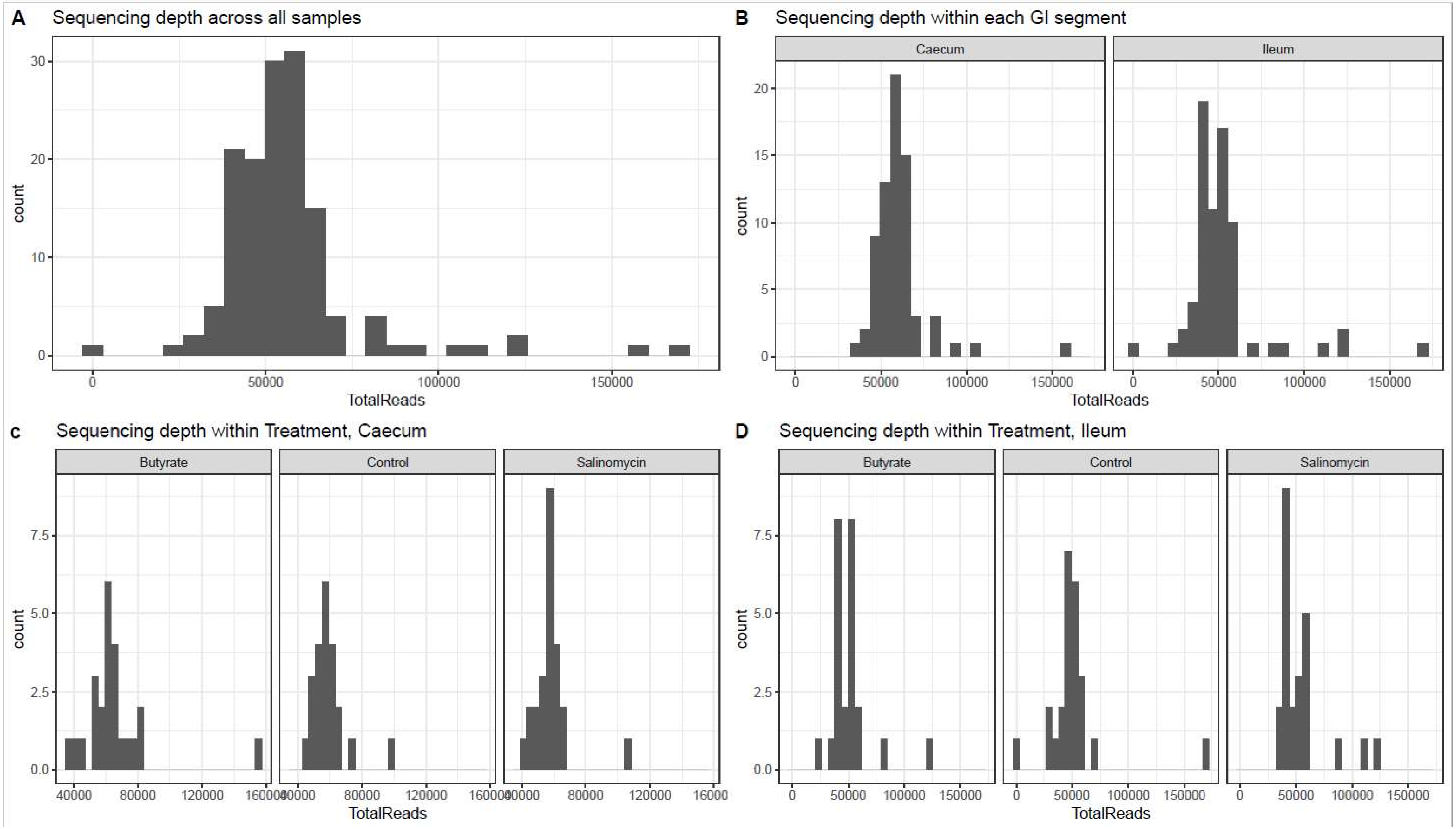

A Sequencing depth across all samples
B Sequencing depth within each GI segment
Caecum
Ileum
c Sequencing depth within Treatment, Caecum
Butyrate
Control
Salinomycin
D Sequencing depth within Treatment, Ileum
Butyrate
Control
Salinomycin
count
TotalReads
