# Supplementary material for: Impact of Dietary Sodium Butyrate and Salinomycin on Performance and Intestinal Microbiota in a Broiler Gut Leakage Model
Source: Animals (Basel). 2022 Jan 4;12(1):111. doi: 10.3390/ani12010111 (PMC8749775; doi:10.3390/ani12010111)
Supplement: Supplementary file 1 [file animals-12-00111-s001.zip › Supplementary Figure S2_Rarefaction curves of ASVs clustered at 99% sequence identity.pptx]

## Slide 1
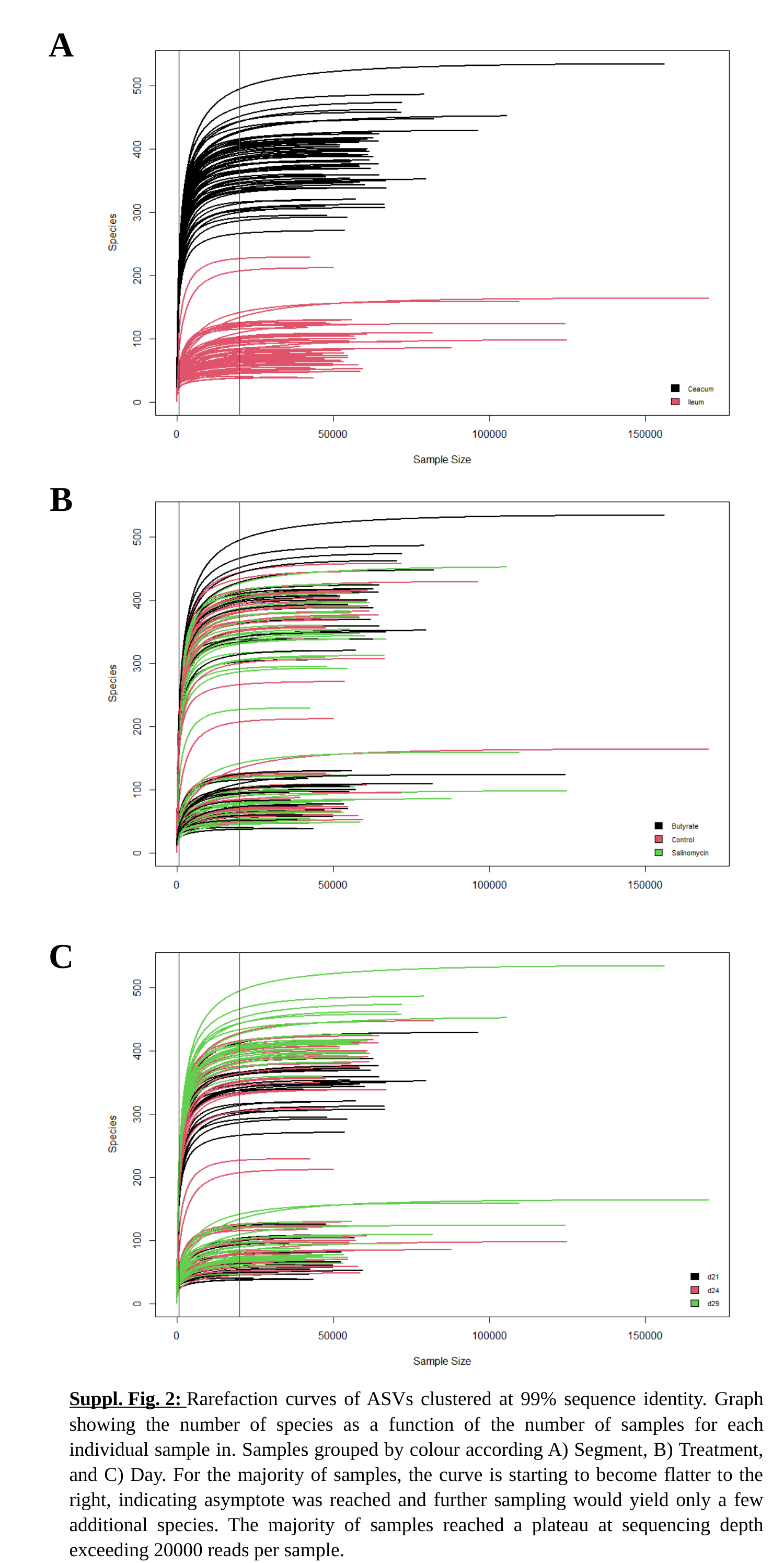

A
B
C
Suppl. Fig. 2: Rarefaction curves of ASVs clustered at 99% sequence identity. Graph showing the number of species as a function of the number of samples for each individual sample in. Samples grouped by colour according A) Segment, B) Treatment, and C) Day. For the majority of samples, the curve is starting to become flatter to the right, indicating asymptote was reached and further sampling would yield only a few additional species. The majority of samples reached a plateau at sequencing depth exceeding 20000 reads per sample.
