# Supplementary material for: Impact of Dietary Sodium Butyrate and Salinomycin on Performance and Intestinal Microbiota in a Broiler Gut Leakage Model
Source: Animals (Basel). 2022 Jan 4;12(1):111. doi: 10.3390/ani12010111 (PMC8749775; doi:10.3390/ani12010111)
Supplement: Supplementary file 1 [file animals-12-00111-s001.zip › Supplementary Table S1 Diet composition.pdf]

Table S1. Ingredient and calculated nutrient composition (g/kg) of starter diets (days 1 to 10) and grower diets (days 10 to 35)

Ingredient (g/kg)	Starter			Grower		
	Control	Butyrate	Salinomyin	Control	Butyrate	Salinomycin
Wheat	475.7	475.3	475.5	635.2	635.9	634.8
Maize	100.0	100.0	100.0	-	-	-
Rye	-	-	-	50.0	50.0	50.0
Soybean meal	285.0	285.0	285.0	213.0	213.0	213.0
Rape seed (LL)	50.0	50.0	50.0	55.0	54.0	55.0
Oat	25.0	25.0	25.0	-	-	-
Soya oil	15.0	15.0	15.0	10.0	10.0	10.0
Monocalcium phosphate	17.3	17.4	17.3	12.5	12.5	12.5
Limestone	12.2	12.0	9.4	9.8	9.6	7.0
Sodium bicarbonate	2.8	2.8	2.8	2.0	1.9	2.0
Sodium chloride	1.7	1.7	1.7	1.8	1.9	2.0
DL-methionine	3.6	3.6	3.6	2.9	2.9	2.9
Lysine HCl	6.0	6.0	6.0	3.0	3.0	3.0
L-Threonine	2.2	2.2	2.2	1.8	1.8	1.8
Vitamin and Mineral mix[1]	3.5	3.5	3.5	3.0	3.0	3.0
Salinomycin premix[2]	-	-	3.0	-	-	3.0
Butyrate coated[3]		0.5			0.5	
Calculated nutrients (g/kg)						
Dry matter	883.3	883.5	883.3	882.5	882.4	882.4
Crude protein	224.4	224.3	224.4	200.3	200.2	200.3
Crude fat	61.2	61.3	61.2	55.5	55.2	55.5
Ash	55.9	56.0	56.3	45.9	45.9	46.3
Starch	346.9	346.7	346.8	386.7	387.1	386.6
Crude fibre	30.1	30.1	30.1	27.6	27.5	27.6
Total calcium	9.60	9.60	9.61	7.65	7.63	7.66
Total phosphorus	7.65	7.68	7.66	6.40	6.40	6.40
Available phosphorus	4.80	4.82	4.80	3.80	3.80	3.80
Metabolizable energy (kcal/kg)	2859	2860	2859	2876	2875	2875

[1]Supplied per kilogram of starter diet: Vitamin A, 12,000 IU; Vitamin D, 5,000 IU; Vitamin E, 54 IU; Vitamin B12, 0.016 mg; Riboflavin, 6.00 mg; Niacin, 55.00 mg; d-Pantothenic acid, 13.00 mg; Menadione, 2.99 mg; Folic acid, 3.05 mg; Pyroxidine, 4.00 mg; Thiamine, 2.00 mg; Biotin, 0.20 mg; Betaine hydrochloride, 260.00 mg; Manganese dioxide, 120.00 mg; Zinc oxide, 100.00 mg; Iron sulfate, 20.00 mg; Copper sulfate, 18.00 mg; Calcium iodate, 2,00 mg; Sodium selenite, 0,30 mg. The carrier was calcium carbonate.

Supplied per kg of grower diet: Vitamin A, 10,286 IU; Vitamin D, 4,286 IU; Vitamin E, 47 IU; Vitamin B12, 0.013 mg; Riboflavin, 5.14 mg; Niacin, 47.14 mg; d-Pantothenic acid, 11.14 mg; Menadione, 2.57 mg; Folic acid, 2.61 mg; Pyroxidine, 3.43 mg; Thiamine, 1.71 mg; Biotin, 0.17 mg; betaine hydrochloride, 222.86 mg; Manganese dioxide, 102.86 mg; Zinc oxide, 85.71 mg; Iron sulfate, 17.14 mg; Copper sulfate, 15.43 mg; Calcium iodate, 1.71 mg; Sodium selenite, 0.26 mg.

[2] The Salinomycin premix (Sacox®, Huvepharma, Bulgaria) contained calcium carbonate as a carrier and supplied per kg of starter and grower diet 69 mg salinomycin.

[3] The coated butyrate was UltraGuard™-DUO, a combination of protected sodium and calcium butyrate, Devenish, Devenish Nutrition Limited (https://www.devenishnutrition.com/about/devenish-group/
