# Supplementary material for: Impact of Dietary Sodium Butyrate and Salinomycin on Performance and Intestinal Microbiota in a Broiler Gut Leakage Model
Source: Animals (Basel). 2022 Jan 4;12(1):111. doi: 10.3390/ani12010111 (PMC8749775; doi:10.3390/ani12010111)
Supplement: Supplementary file 1 [file animals-12-00111-s001.zip › Supplementary Table S2.pdf]

Table S2 PERMANOVA results of ASVs abundance data from cacal samples on days 21, 24 and 29 using the Bray–Curtis dissimilarity matrix with 999 permutations.

Treatment	df	SS	F Model	R^2	P-adjusted
**Day 21**					
**Diet (F2,21 = 1.13, P = 0.09)**					
Control_vs_Salinomycin	1	0.17024	1.3808	0.08977	0.026
**Day 24**					
**Diet (F2,21 = 1.97, P = 0.001)**					
Salinomycin_vs_Butyrate	1	0.27413	2.863	0.16978	0.001
Salinomycin_vs_Control	1	0.16689	1.6052	0.11798	0.021
**Day 29**					
**Diet (F2,21 = 1.93, P = 0.001)**					
Control_vs_Salinomycin	1	0.20456	1.9526	0.1224	0.002
Butyrate_vs_Salinomycin	1	0.29239	2.8577	0.16952	0.001

Pairwise comparisons used Bonferroni correction. A p-value ≥ 0.05 is statistically significant.
