# Supplementary figures and images for: Impact of Dietary Sodium Butyrate and Salinomycin on Performance and Intestinal Microbiota in a Broiler Gut Leakage Model
Source: Animals (Basel). 2022 Jan 4;12(1):111. doi: 10.3390/ani12010111 (PMC8749775; doi:10.3390/ani12010111)

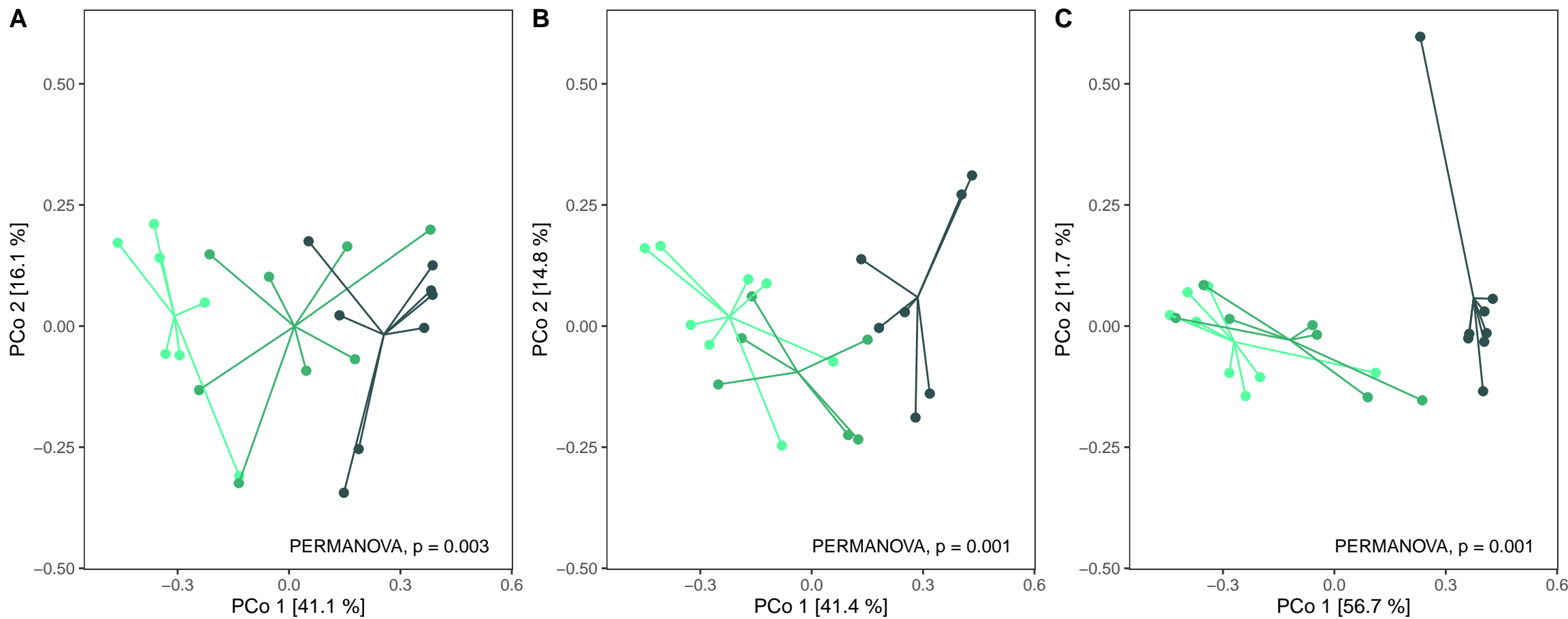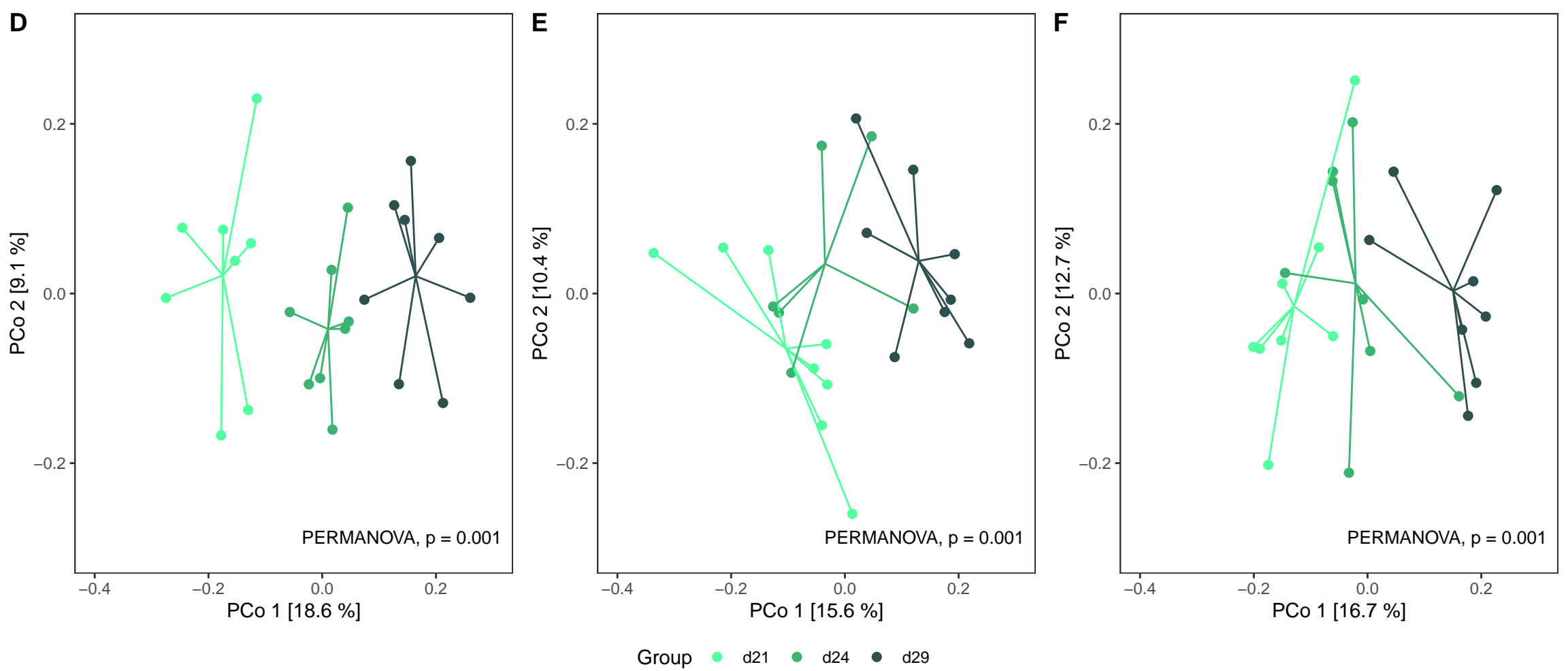

Supplement: Supplementary file 1 [file animals-12-00111-s001.zip › Supplementary Figure S3_BrayCurtis_Ordination_All_DayperTreatment_20210505_update.pdf]

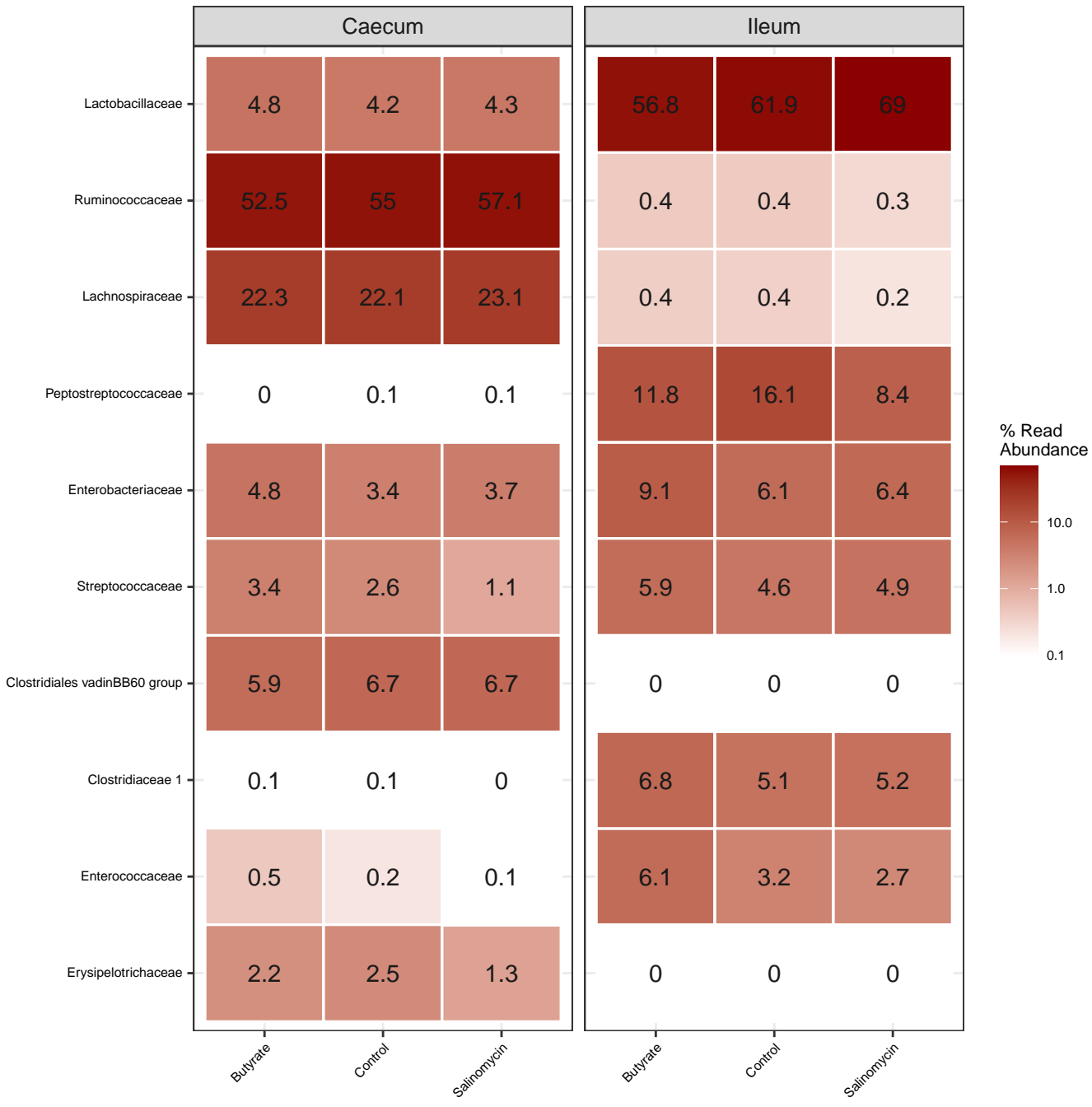

Supplement: Supplementary file 1 [file animals-12-00111-s001.zip › Supplementary Figure S4_Top10AbundantFamilies_acrossAllSamples_20210315.pdf]
